# Microstructure Evolution and Strengthening Mechanism of Dual-Phase Mg–8.3Li–3.1Al–1.09Si Alloys during Warm Rolling

**DOI:** 10.3390/ma17102321

**Published:** 2024-05-14

**Authors:** Ying Wang, Guangying Wu, Bingbing Liang, Yongquan He, Changhong Liu, Junwei Liu, Guobing Wei

**Affiliations:** 1 School of Materials Science and Engineering, Zhengzhou University of Aeronautics, Zhengzhou 450046, China; 2 International Joint Laboratory for Light Alloys, Ministry of Education, Chongqing University, Chongqing 400044, China

**Keywords:** Mg–Li alloy, Mg_2_Si phase, microstructure evolution, strengthening mechanism, warm rolling

## Abstract

In this study, the rolling process of the warm-rolled duplex-phase Mg–8.3Li–3.1Al–1.09Si alloy and the strengthening mechanism of as-rolled Mg–Li alloy were investigated. The highest ultimate tensile strength (UTS, 323.66 ± 19.89 MPa) could be obtained using a three-pass rolling process with a 30% thickness reduction for each pass at 553 K. The strength of the as-rolled LAS831 alloy is determined by a combination of second-phase strengthening, grain refinement strengthening, dislocation strengthening, and load-transfer reinforcement. Of these factors, dislocation strengthening, which is caused by strain hardening of the α-Mg phase, can produce a good strengthening effect but also cause a decrease in plasticity. The Mg_2_Si phase is broken up into particles or strips during the rolling process. After three passes, the AlLi particles were transformed into an AlLi phase, and the Mg_2_Si particles and nanosized AlLi particles strengthened the second phase to form a hard phase. The average size of the DRXed β-Li grains decreased with each successive rolling pass, and the average size of recrystallized grains in the three-pass-rolled LAS831 alloy became as low as 0.27 μm. The interface between the strip-like Mg_2_Si phase and the α-Mg phase is characterized by semicoherent bonding, which can promote the transfer of tensile and shear forces from the matrix to the strip-like Mg_2_Si phase, thereby improving the strength of the matrix and thus strengthening the LAS831 alloy.

## 1. Introduction

As the lightest structural metal materials, Mg–Li alloys have recently attracted increased interest for both scientific research and industrial applications due to their high specific strength, good formability, excellent damping, and good mechanical properties at room temperature [1,2], and these materials are expected to have great application potential in the aerospace, weapon, and 3C (Computer, Communication, Consumer Electronics) industries. According to the Mg–Li binary phase diagram [3,4], structural transformation occurs as the Li content is increased in Mg–Li alloys. In Mg–Li alloys, α-Mg is maintained in a hexagonal close-packed (HCP) structure when the lithium content is below 5.7 weight percent (wt%), which is indicative of limited formability and moderate strength. When the Li content exceeds 10.3 wt%, β-Li phases with body-centered cubic (bcc) structures are formed, which effectively improves the formability, but the strength is significantly reduced. Between 5.7 and 10.3 wt% Li, there is phase transformation from a bcc structure (β-Li) into an HCP structure (α-Mg), and a combination of α-Mg and β-Li coexist in the microstructure. Due to the desirable mechanical properties resulting from the various phases and phase-to-phase interactions, Mg–Li alloys exhibit interesting mechanical properties by combining the excellent ductility of the β phase with the moderate strength of the α phase [5]. However, their poor thermal stability and lower absolute strength limit their application [6]. To resolve these problems, researchers have applied different processing methods, such as grain refinement, severe plastic deformation, alloying, and heat treatment [7]. In these methods, the addition of traditional elements (such as Zn [8] and Al [9]) and rare earth (RE) [10] elements is an effective and relatively simple approach. However, the UTS of most Mg–Li alloys strengthened by alloying and severe plastic deformation (SPD), such as equal channel angular pressing (ECAP), still can barely surpass 200 MPa [6,7,8,9,10].

Si is an effective alloying element that, together with Mg, can form the intermetallic compound Mg_2_Si, which is characterized by a high melting point (1358 K), low density (1.99 g/cm^3^), high hardness (4.5 × 10^9^N/m^2^), high Young’s modulus (120 GPa), and low thermal expansion coefficient. Therefore, the presence of a Mg_2_Si phase can greatly improve the strength and thermal stability of Mg–Li alloys without increasing their density. The results from a few recently published papers on Si-containing Mg–Li alloys [11] show that their strength is greatly improved. This improvement of mechanical properties with the addition of Si is attributed to the precipitation hardening effect of the Mg_2_Si precipitates. The strength of alloys significantly increases with increasing Si content [12]; however, when the Si content exceeds 1.14 wt%, coarse dendritic Mg_2_Si phases appear, which are not desirable because they split the alloy matrix and considerably deteriorate the mechanical properties [13]. Moreover, the plastic forming ability of the alloy greatly decreases. Therefore, the Si content in magnesium–lithium alloys currently remains low, which is not conducive to the development of magnesium–lithium alloys with increased strength. For this reason, several techniques have been applied in refining the coarse Mg_2_Si phases of Si-containing Mg alloys, including rapid solidification, ultrasonic treatment, plastic deformation, and the addition of alloys [14,15].

The supergravity and vibration inherent in centrifugal casting technology accelerate solidification of the melt and refine Mg_2_Si particles in aluminum–silicon alloys [16]. Moreover, the self-purification of melts, which occurs under the action of supergravity during the solidification process of centrifugal casting, can promote the phase separation of inclusions and coarse primary Mg_2_Si in melts, improving the quality of ingot formation. Purified ingots containing refined Mg_2_Si particles represent a good foundation for the deformation processing of alloys. The alloy is subsequently rolled, wherein the Mg_2_Si phase particles are crushed via large deformation.

Therefore, in this study, Mg–8.3Li–3.1Al–1.09Si (LAS831) alloy ingots were prepared using centrifugal casting, and the defective parts at the top and bottom of the ingot were then removed via machining. Finally, LAS831 alloy sheets were prepared using a multipass rolling process. The microstructural evolution and strengthening mechanism of the LAS831 alloy during the deformation process are investigated.

## 2. Materials and Methods

In this study, the experimental alloys used are commercial pure Mg ingot (99.7%), pure Li strips (99.9%), pure Al ingot (99.8%), and Mg–10Si master alloy (all in wt.%, unless otherwise stated). The as-cast LAS831 alloy was prepared by smelting. First, the pure Mg ingot was placed into a high-purity graphite crucible and heated to 993 K under the protection of Ar gas until melting. Then, certain amounts of Mg–10Si intermediate alloy and Al ingots were added to the Mg melt, heated to 1123 K, kept warm, and stirred for 5–10 min. The crucible was then cooled to 993 K, a given mass of pure Li strips was weighed and quickly wrapped in aluminum foil, and the wrapped lithium ingot was quickly put into the melt and mechanically stirred for 5–10 min. Finally, the melt was poured into a mold under a pure argon atmosphere and mounted onto a centrifugal casting machine in the temperature range of 963 K–973 K. Subsequently, the centrifugal casting machine drive motor was turned on, the centrifugal casting machine speed was adjusted to 600 r/min, and the machine speed was maintained for 10 min. Then, the drive motor was turned off, and the casting was removed. Machining was used to remove defective parts such as the heads and tails of the casting. After machining, the ingots were cut into 120 mm × 80 mm × 10 mm sheets and annealed at 573 K for 60 min. Before the rolling process, the alloy sheets were heated at 553 K for 30 min. The reduction per rolling pass is 30 % of the sheet thickness. After one-pass rolling, the thickness of the alloy sheet is reduced from 10 mm to 7 mm; after two-pass rolling, the thickness of the alloy sheet is reduced from 7 mm to 4.9 mm; after three-pass rolling, the thickness of the alloy sheet is reduced from 4.9 mm to 3.43 (≈3.5) mm. Rollers with a diameter of Ø220 mm were preheated to 553 K, and the temperatures of alloy sheets and rollers during the rolling process were monitored using an infrared temperature tester.

The actual chemical composition of the LAS831 alloy was tested using inductively coupled plasma optical emission spectrometry (ICP-OES, Agilent 5800, Santa Clara, CA, USA), and the results are listed in Table 1. The microstructure was examined using optical microscopy (OM, Olympus GX-51, San Jose, CA, USA) after etching with oxalic solution (1.5 g of oxalic acid, 1 mL of acetic acid, and 97.5 mL of H_2_O). The microstructure observation was carried out with samples positioned perpendicular to the normal direction (ND)–rolled direction (RD) of the rolled sheets. Transmission electron microscopy (TEM; JEOL 2100, Tokyo, Japan) was performed in conjunction with energy-dispersive X-ray spectroscopy (EDS; Oxford X-MAX20, Oxford, UK). Thin foil specimens for TEM observation were prepared by wire electrical discharge cutting into 0.2 mm thin slices along the ND–RD direction of the rolled sheets, and then hand force grinding to 50 μm thick, followed by low-temperature (liquid nitrogen) argon ion milling to 50 nm thick. The phase composition was identified using an X-ray diffractometer (XRD; Rigaku Smart Lab, Tokyo, Japan) and Cu–Kα radiation with a scanning rate of 10 deg/min, which was carried out perpendicular to the ND–RD of the rolled sheets.

**Figure 1:**
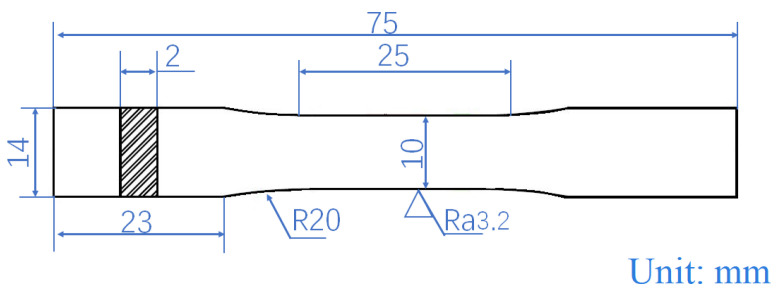
The detailed dimensions of the dog-bone-shaped tensile samples.

The dimensions of tensile samples are length of 25 mm, width of 10 mm, and thickness of 2 mm, where the length corresponds to the direction of rolling, and the detailed dimensions of the samples are shown in Figure 1. The tensile tests were conducted following the ASTM E8/E8M-21 standard [17], and testing of each rolled sheet was repeated three times. The tensile tests were carried out using a CMT-5205 electronic universal testing machine (SAS Test, Shenzhen, China) at room temperature with an initial strain rate of 0.5 mm/min.

## 3. Results and Discussion

### 3.1. Phase Analysis

**Figure 2:**
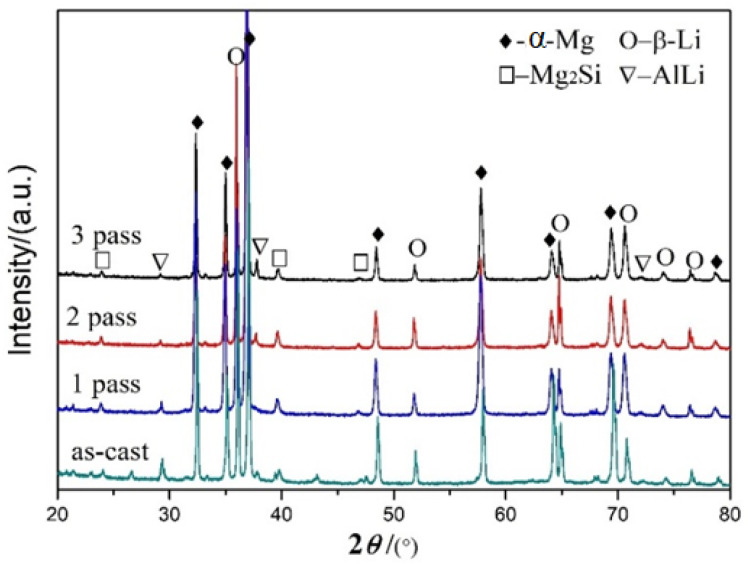
XRD pattern of the LAS831 alloy after each rolling pass.

The XRD patterns of the LAS831 alloys after different numbers of rolling passes were determined via XRD mapping analysis (Figure 2). The alloys were found to primarily comprise a dual phase of HCP (α-Mg) + bcc (β-Li); in addition, a small part of the alloy comprises AlLi and Mg_2_Si phases, as shown in Figure 2. With each successive rolling pass, the phase composition of the alloy does not change, except for some peak strength changes.

### 3.2. Microstructure

**Figure 3:**
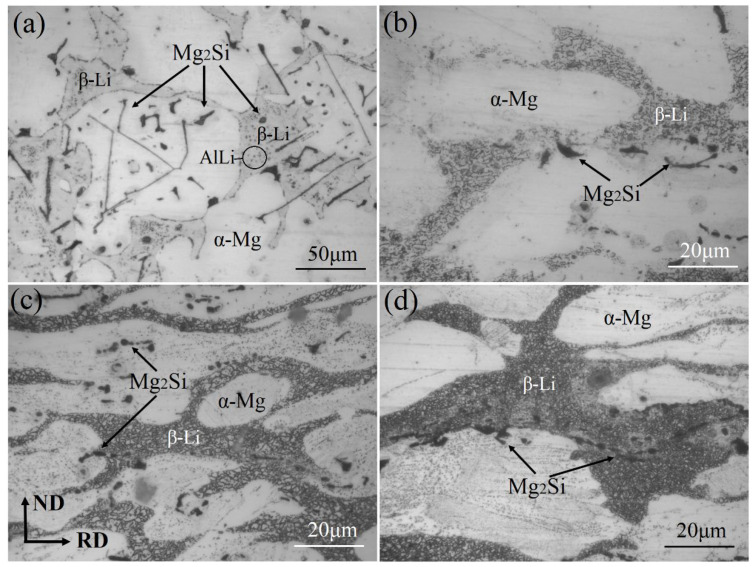
Optical microstructure of the LAS831 alloy: (**a**) as-cast, (**b**) one-pass rolled, (**c**) two-pass rolled, and (**d**) three-pass rolled.

Optical micrographs of the as-cast and ND–RD planes of the alloys as-rolled with different numbers of rolling passes are shown in Figure 3. Figure 3a shows the microstructure of the centrifugally cast LAS831 alloy. The LAS831 alloy has a typical duplex phase (the light HCP α-Mg and gray bcc β-Li) structure and black Mg_2_Si phase. The lamellar and granular black Mg_2_Si phases are distributed in the matrix phase. Some small spherical particles with an average size of about 1 μm are uniformly distributed in the β phase and the α/β phase boundaries of the LAS831 alloy. According to the XRD results shown in Figure 2, the particles are likely the Al–Li phase.

As shown in Figure 3, both β-Li and α-Mg phases are elongated during the rolling process. The microstructure of several flat α-Mg phases illustrates the slip trace patterns during the rolling procedure. The β-Li phase recrystallizes during the rolling process, and the dynamic recrystallization (DRX) grain size of the β-Li phase decreases gradually with the increasing number of rolling passes. After three rolling passes, the recrystallization grain size of the β-Li phase became even smaller than the resolution of the optical microscope. The lamellar and granular black Mg_2_Si phases were broken during rolling deformation; furthermore, with increasing rolling pass, the particle size of the Mg_2_Si phases gradually increased, and the length of the strip-like Mg_2_Si gradually increased along the rolling direction.

### 3.3. TEM Analysis

TEM is the main means for evaluating nanograins and substructures because it can obtain direct images of nanocrystals and substructures. Microstructure evolution during the rolling process was further studied via TEM images of the different rolling passes sheets.

**Figure 4:**
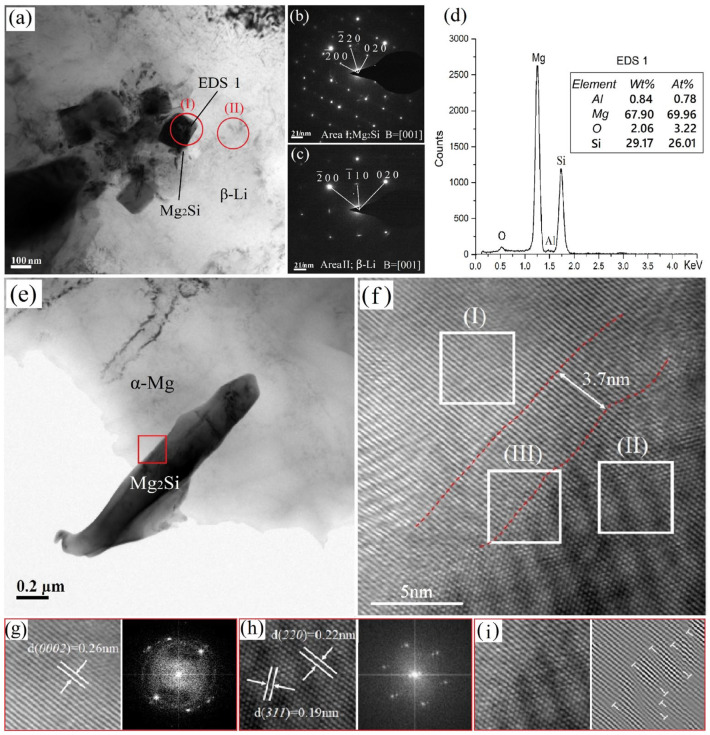
TEM characterization of the three-pass-rolled LAS831 alloy: (**a**) TEM image of Mg_2_Si grain-shaped nanoparticles; (**b**) and (**c**) the corresponding SAED patterns of the red-circled areas (I) and (II) shown in (**a**); (**d**) TEM-EDS elemental mapping of the red-circled area (I) shown in (**a**); (**e**) TEM image of the stripe-shaped Mg_2_Si; (**f**) HR-TEM images of the Mg_2_Si/α-Mg interface in the red-square area shown in (**e**); FFT image and characteristic spacings for the lattice planes of (**g**) Mg_2_Si (area (I) in (**f**)) and (**h**) α-Mg (area (II) in (**f**)); and (**i**) inverse FFT images of the Mg_2_Si/α-Mg interface of area (III) shown in (**b**).

The TEM images of rolled LAS831 alloy after three passes were shown in Figure 4. Two types of second phases can be observed in Figure 4a,e: grain-shaped and stripe-shaped. Based on the XRD test results and the previous literature, the second phase is identified as Mg_2_Si. As shown in Figure 4a, grain-shaped nanoparticles are distributed in the white matrix, and the corresponding SAED patterns of red-circled area I and red-circled area II are shown in Figure 4b and Figure 4c, respectively. The SAED pattern (Figure 4c) from the [0 0 1] β direction of the β-Li matrix demonstrated that the matrix had a BCC structure, which was confirmed to be the β-Li phase. The SAED patterns (Figure 4b) were analyzed and reveal a face-centered cubic (fcc) structure with space group Fm3m and a zone axis B = [0 0 1]. The EDS spectrum (Figure 4d) shows that the main elements of the grain-shaped particulate phase are Mg and Si, at an atomic ratio of close to 2:1, and the Mg_2_Si phase was identified. In addition, the SAED pattern in Figure 4b confirms that the Mg_2_Si phase has the following orientation relationship with the matrix: [0 0 1] β//[0 0 1] Mg_2_Si. Figure 4e shows a strip-like phase embedded in the matrix, the HR-TEM images of the red-square area are shown in Figure 4f, and the bacilliform phase/matrix interface is visible in the HR-TEM image in Figure 4e. At the interface, a layer ~3.7 nm in thickness can be observed. Lattice fringes can be clearly observed in area (I) of Figure 4f. The lattice planes (0002) of α-Mg can be determined after fast Fourier transform (FFT), as shown in Figure 4g. Lattice fringes can be clearly observed in area (II) of Figure 4f. After FFT, the (220) and (311) lattice planes of Mg_2_Si can be determined accordingly, as shown in Figure 4h. Figure 4i is the lattice fringe image obtained using FFT, and filtering and inverse FFT for the squareness area are marked by III in Figure 4f, where the partial misfit dislocations (white T-shape) are also marked. The relationship between the Mg_2_Si phase and the α-Mg matrix is semicoherent.

**Figure 5:**
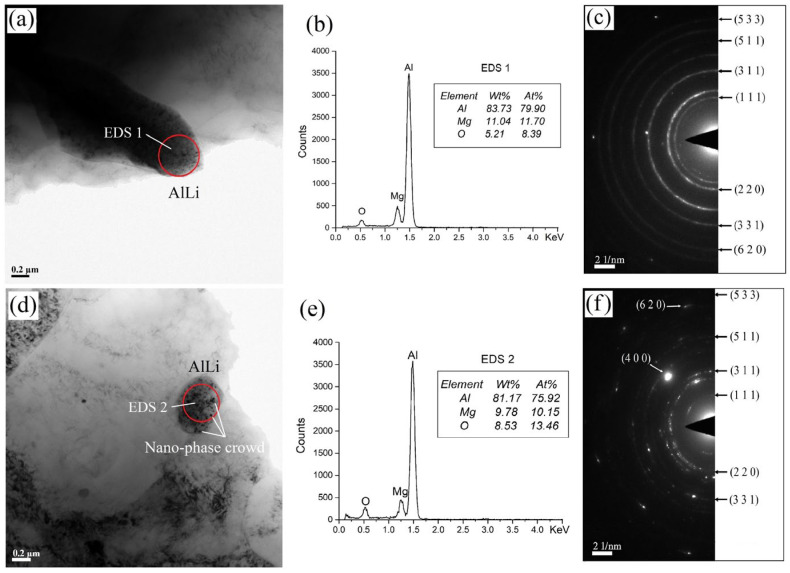
TEM characterization of the as-rolled LAS831 alloy: (**a**) AlLi phase in two-pass-rolled LAS831; (**b**,**c**) TEM-EDS energy spectrum and SAED patterns of the red-circled area shown in (**a**); (**d**) AlLi phase in three-pass-rolled LAS831; (**e**,**f**) TEM-EDS elemental mapping and corresponding SAED patterns of the red-circled area shown in (**d**).

Because Li is too light for this analysis, the AlLi phase can instead be determined by combining TEM data and XRD patterns. The interference of spherical precipitate with the matrix in the two-pass- and three-pass-rolled LAS831 alloys was analyzed via TEM, as shown in Figure 5. The red-circled area in Figure 5a was subjected to EDS testing and SAED pattern shooting, and the results are shown in Figure 5b and Figure 5c, respectively. The main characteristic X-rays of Al are observed in the EDS spectrum (Figure 5b), and the atomic percentage (At%) of aluminum in the spherical precipitate is as high as 79.90%, so the spherical precipitate was initially identified as AlLi phase.

As shown in Figure 5c, the continuous diffraction rings in the SAED pattern indicate the presence of randomly crystallographic orientated nanograins. Dislocation slip and deformation twinning are activated during the rolling process, and the number of dislocations increases with the increase in strain, resulting in the coarse grains being divided into substructures. Recrystallization promotes the transformation of subgrain boundaries into highly mismatched grain boundaries, which results in the formation of nanograins [18]. In this study, the halo pattern in the diffraction profile also indicates that the nanocrystalline phase is formed after two rolling passes, as demonstrated in Figure 5c, which matched with the d-values of the (111), (220), (311), (331), (511), (620), and (533) planes of AlLi. With each successive rolling pass, the numerous dislocations accumulate, and a large number of deformation dislocations are absorbed via nanophase recovery and recrystallization, which promotes the migration of nuclei and boundaries. The nanograin AlLi phase grew near the region where the dislocations accumulate, which is consistent with the observation in Figure 5d. The crystal lattice (400) and (620) planes, shown by the SAED pattern (Figure 5f), also illustrate this phenomenon of nanograin growth.

**Figure 6:**
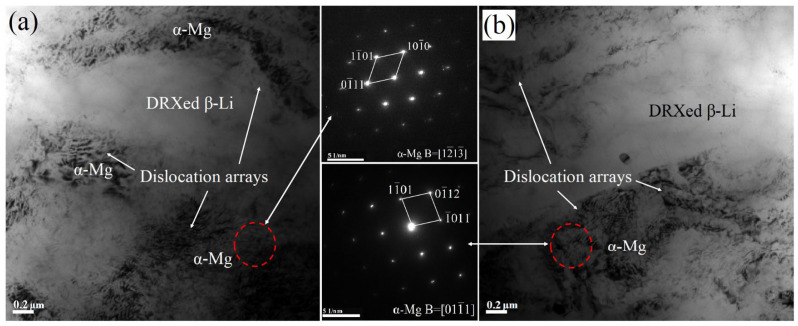
TEM characterization of the as-rolled LAS831 alloy. TEM image of the rolled LAS831 alloy and SAED pattern of α-Mg for (**a**) two-pass rolling and (**b**) three-pass rolling.

Figure 6 shows the detailed substructures of the LAS831 sheets. According to the SAED pattern calibration, the deep-colored phases are α-Mg phases while the light-colored phases are β-Li phases. Because the softer β-Li phase preferentially undergoes DRX compared to the α-Mg phase, a large amount of dense dislocation is consumed in the dynamic recrystallization process of the β-Li phase, leading to a lighter color of the β-Li phase [19]. Due to incomplete dynamic recrystallization of the α-Mg phase during the rolling process, the fiber structure is maintained along the direction of rolling. In the process of deformation, grains that were not dynamically recrystallized (DRXed) elongate along the direction of deformation [20].

**Figure 7:**
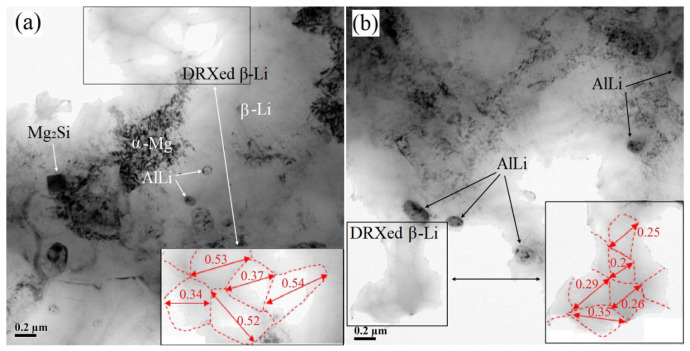
TEM characterization of the as-rolled LAS831 alloy. TEM image of the rolled LAS831 alloy and grain size of DRXed β-Li for (**a**) two-pass rolling; (**b**) three-pass rolling.

Figure 7 shows some examples of DRXed grains in the β-Li phase. The grains have sharp angles and straight boundaries, and the organization of the grain boundaries is uniform and clean. The fine equiaxed DRXed grains are formed in the microstructure of the alloy after two-pass rolling (Figure 7a), and the average size of DRXed grains (calculated from five grains) of the β-Li phase is ~0.46 μm. The average DRXed grain size (calculated from five grains) of the β-Li phase of the three-pass-rolled alloy is ~0.27 μm (Figure 7b). The three-pass-rolled samples have an obviously smaller recrystallization grain size than the two-pass-rolled samples. The main reason is that DRX occurs during the third rolling process, which results in a decrease in the grain size.

### 3.4. Mechanical Properties

**Figure 8:**
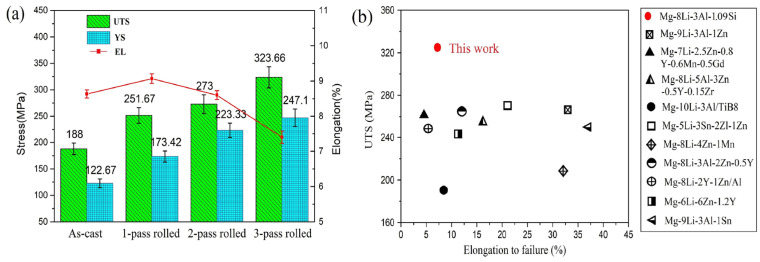
(**a**) Tensile test results of LAS831 with different numbers of rolling passes. (**b**) Tensile properties of Mg–Li alloys [6,21,22,23,24,25,26,27,28,29].

Figure 8 shows the tensile test results for the LAS831 alloys after different numbers of rolling passes. The tensile properties of the materials are shown in Figure 8a. The UTS of centrifugal casting LAS831 alloy reach 188 ± 10.3 MPa (mean ± square error) and an elongation to failure (EL) of 8.63 ± 0.14%. The alloy UTS increased during the rolling procedure. With each successive rolling pass, both the UTS and yield strength (YS) increased, and the highest YS and UTS values of 247.1 ± 13.67 MPa and 323.66 ± 19.89 MPa, respectively, were obtained for three-pass-rolled alloy. The EL decreases with the rolling passes increasing. The lowest EL of 7.4 ± 0.17% was obtained in three-pass-rolled alloy. Compared with other recent studies (Figure 8b), the tensile strength of the dual-phase LAS831 alloy in this work is significantly higher than that in other studies. The strength of Mg–Li alloy is mainly a result of plastic deformation, which refines grains and introduces high-density dislocations, among other factors. In our work, a suitable warm-rolled LAS831 alloy was explored, and a UTS of up to 323.66 ± 19.8 MPa was ultimately obtained using a three-pass 30% reduction rolling process. It can be observed that compared with the Mg-Li alloys containing rare earth alloy, the specific strength and absolute strength of the alloy in this study are advantageous.

**Figure 9:**
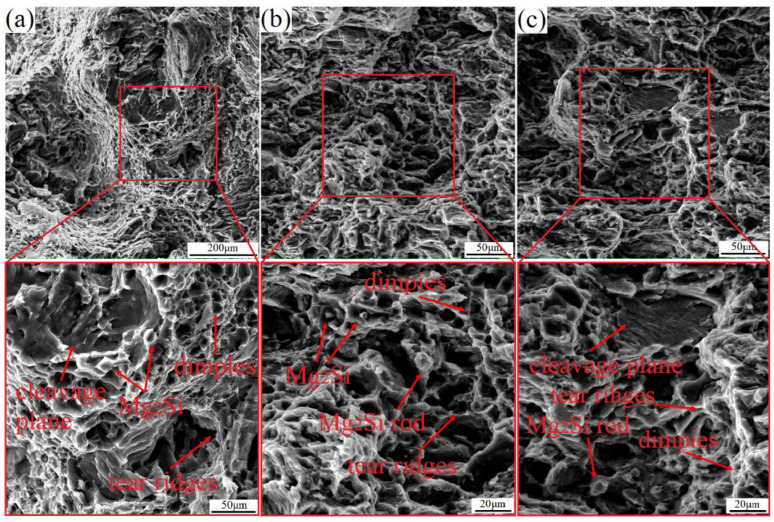
Microstructure of the fracture surfaces of the as-cast and as-rolled LAS831 alloys with different numbers of rolling passes: (**a**) as-cast, (**b**) one pass, and (**c**) three passes.

In general, the plasticity of the matrix phase and the morphology of the reinforcing phase strongly influence the elongation to fracture of an alloy, and the size and depth of the dimples increase with increasing plasticity during fracturing of the material. The fracture of magnesium alloys is brittle and exhibits cleavage and quasicleavage modes due to the α-Mg phase and the HCP structure. Figure 9 shows the fracture morphology of the as-cast and as-rolled LAS831 alloys with different numbers of rolling passes. As shown in Figure 8a, there is an obvious cleavage plane, and additional small dimples can be observed at the fracture surface of the as-cast specimen. Figure 3a shows that there is a large number of lamellar Mg_2_Si phases in the as-cast alloy. Due to the substantial difference in the elastic modulus between the α-Mg phase and the Mg_2_Si phase, a large amount of strain is concentrated at the α-Mg/Mg_2_Si phase boundary during tensile deformation of the sample, which eventually leads to the appearance of a cleavage plane. The dimples mainly appear at the fracture position of the β-Li phase. As the lamellar Mg_2_Si phase breaks during rolling deformation, stress concentrations cannot accumulate at the α-Mg/Mg_2_Si interface, and no cleavage planes are found in the fracture morphology of the one-pass-rolled sample. With the increase in rolling passes, the α-Mg phase accumulates a large amount of strain strengthening, and a cleavage plane is found in the fracture morphology of the three-pass-rolled samples. The appearance of the cleavage plane in the fracture morphology indicates that the elongation to fracture of the alloy decreased. This conclusion is consistent with the tensile test results.

## 4. Discussion

### 4.1. Influence of Rolling Passes on the Microstructure Evolution of LAS831 Alloy

According to Figure 3a, there are some lamellar and granular Mg_2_Si phases in the as-cast LAS831 alloy. These bulky second phases easily became a source of cracks during the tensile test. Therefore, the strength and fracture plasticity remain low. In Figure 3b–d, the Mg_2_Si phase is mainly grain-shaped and evenly distributed, representing a hardening phase that is good for enhancing strength. With each successive rolling process, the size of the Mg_2_Si phase gradually decreases.

The changes in microstructure caused by rolling processing can be explained on the basis of the comprehensive effects of rolling strain and dynamic recrystallization. Grain crushing, rolling stress, and DRX occurred at the same time during warm rolling. After one rolling pass with a thickness reduction of approximately 30%, there is an increase in the effects of DRX of β-Li, grain crushing of Mg_2_Si, and rolling stress of the α-Mg phase. Consequently, as exhibited in Figure 3 and Figure 6, the α-Mg phase becomes obviously elongated, and dislocation density increases due to rolling strain during deformation. There was no observed recrystallization of α-Mg in the alloy microstructure with the different numbers of rolling passes. The main reason is that the temperature required for α-Mg recrystallization is usually greater than 573 K, and the rolling temperature is only 553 K. Because of its HCP structure, the α-Mg phase has a relatively lower stacking fault energy (SFE), and dynamic recovery of the α-Mg phase is difficult [30]. Therefore, it is difficult to eliminate strain hardening of the α-Mg phase via dynamic recovery, which increases with the increasing number of rolling passes. Thus, while the fracture plasticity decreases, the alloy strength increases.

As shown in Figure 3 and Figure 7, the complete dynamic recrystallization of β-Li phases can be observed. The β-Li phase has relatively higher stacking fault energy (SFE) due to its bcc structure. A higher SFE is beneficial for dynamic recovery and dynamic recrystallization. Thus, dynamic recovery of the β-Li phase readily occurs during the rolling process, and the release of dislocation entanglement energy promotes the DRX of the β-Li phase [31,32]. Dynamic recrystallization of the β-Li phase occurs during each 30% reduction rolling cycle. The two-pass-rolled sample exhibits a structure of fine equiaxed DRXed grains with an average size of 0.46 μm, and the average size of DRXed β-Li grains of the three-pass-rolled sample reaches as low as 0.27 μm. Therefore, the average size of DRXed β-Li grains decreases with the increasing number of rolling passes.

Dispersion of precipitates is an important strengthening method in addition to strain hardening and fine grain strengthening. During the rolling process, the dispersed AlLi and Mg_2_Si precipitates were broken into small particles and uniformly distributed in the matrix. Clearly, the Mg_2_Si phase is a kind of strengthening phase with high hardness and strong thermal stability. In their study, Sanschagrin et al. [33] also considered that AlLi phases have an ordered B2 structure that is not easy to deform, which indicates that AlLi is a hard phase [34]. Dislocation is hindered by these hard second phases. With partial dislocation, the secondary phase particles are bypassed and moving under the action of greater shear stress, while dislocation rings are formed around the secondary phase particles. The alloy was strengthened under the influence of the hindrance and proliferation of dislocation. Figure 4 and Figure 5 show the TEM microstructure of the two- and three-pass-rolled alloys, respectively. After the rolling process, most of the Mg_2_Si phase solids crushed into the matrix, and there is nanotransformation of the AlLi phase after three rolling passes.

In duplex Mg–Li alloys, the Mg_2_Si phase is usually stronger than the metallic matrix phase. Figure 4e,i show that the interface between the strip-like Mg_2_Si phase and the metallic matrix α-Mg phase is characterized by semicoherent bonding. Semicoherent bonding is a strong bonding interface. The stress and strain produced by the alloy load can be passed on to the strip-like Mg_2_Si phase via the metal matrix, which endows this alloy with higher strength.

### 4.2. Strengthening Mechanisms

As shown in Figure 8, the number of rolling passes has an obvious effect on the mechanical properties of the LAS831 sheets. The strength of the as-rolled LAS831 alloy significantly increased after the rolling process. The highest UTS (323.66 ± 19.8 MPa) was obtained using a three-pass rolling process. As a typical duplex-phase alloy, microstructural changes in the matrix phase (α-Mg and β-Li) during the rolling process can affect the properties of the alloy. According to Figure 3 and Figure 6, the dislocation density of the α-Mg phase greatly increases during the rolling process because the α-Mg phase does not recover or dynamically recrystallize during the deformation process. The strengthening effect of work hardening leads to some degree of poor plasticity. The degree of deformation increases with each successive rolling pass; therefore, the degree of work hardening also increases. With an increasing number of rolling passes, the strength of the alloy increased but the plasticity decreased, which is consistent with the mechanical property test results. In contrast to the matrix of the α-Mg phase, there is complete dynamic recrystallization of another matrix of the β-Li phase during the rolling process, as shown in Figure 3 and Figure 7. The grain size of recrystallized β-Li decreases with increasing deformation degree, which is in accordance with previous reports. The statistical data show that the strength and elongation increase with decreasing grain size. In addition, the morphology and distribution of the second phase in the matrix can also affect the strength and plasticity of the alloy. The bulky Mg_2_Si phase was broken into fine particles or strips during the rolling process. The second phase, which includes the AlLi phase and fine Mg_2_Si phase, hinders the movement of dislocations and enhances the deformation resistance of the alloy [33,34]. The strengthening mechanisms of the as-rolled LAS831 are related to work hardening, DRX, and the change in morphology of the second phase, and can be divided into four types:

(1) Dislocation strengthening

The dislocation strengthening of the as-rolled LAS831 is mainly derived from strain hardening of the α-Mg phase during deformation. According to the Taylor equation, the dislocation strengthening mechanism can be expressed as follows:

$${∆\sigma }_{ds}=a_{p}Gb\rho^{1/2}$$

where $a_{p}$ is a constant, *G* is the shear modulus, *b* is the Burgers vector, and *ρ* is the dislocation density. According to the above equation, when the other parameters remain unchanged, the greater the dislocation density in the alloy, the higher the ${∆\sigma }_{ds}$. In this study, the dislocation density of the α-Mg phase greatly increases during the rolling process, which means that the increase in dislocation density can further improve the strength of the LAS831 alloy.

(2) Grain refinement strengthening

The grain refinement of the as-rolled LAS831 alloy is mainly due to the recrystallization of β-Li during deformation. The contribution of fine grain strengthening to UTS is usually calculated using the Hall–Petch relationship as follows: [35,36],

$${∆\sigma }_{bs}=kd^{-1/2}$$

where *k* is the Hall–Petch coefficient, while *d* is the average grain size. According to the above equation, when the other parameters remain unchanged, the lower the average grain size in the alloy, the higher the ${∆\sigma }_{bs}$. The β-Li phase undergoes complete DRX during the rolling process, and the average grain size of the DRXed β-Li phase in the three-pass-rolled LAS831 alloy reaches as low as 0.27 μm. The ${∆\sigma }_{bs}$ of the alloy increased after rolling process, and furthermore, the strength of LAS831 alloy is improved.

(3) Second-phase strengthening

In the studied alloy, both Mg_2_Si and AlLi with high hardness can hinder the dislocation movement. The dislocation bypass mechanism proposed by Orowan explains the role of hard phases in determining alloy strength. Under the action of shear stress, dislocations cannot directly pass through the hard secondary phases but can continue to bypass the hard phase. The bulky Mg_2_Si phase was broken into fine particles during the rolling process. Moreover, the AlLi phase also underwent nanotransformation. With each successive rolling pass, the degree of deformation increases, and the average radii of Mg_2_Si and AlLi particles decrease. Since the total amount of the second phase remains unchanged during the rolling process, the size of the second phase decreases, resulting in a decrease in the spacing of the second-stage particles. The contribution to the UTS of hard phase strengthening is generally calculated using Morris’s computational model [37], which is as follows:

$${∆\sigma }_{or}=M\frac{0.4Gb}{\lambda }ln\frac{r}{b}$$

where *M* is the mean orientation factor, *λ* is the interparticle distance, *r* is the average radius of the particles, *G* is the shear modulus, and *b* is the magnitude of the Burgers vector. According to the above equation, when the average radius of the particles decreases, the interparticle distance decreases, and ${∆\sigma }_{or}$ increases. In this study, the total volume of AlLi and Mg_2_Si phase in the alloy remains unchanged during the rolling process, and the dispersed AlLi and Mg_2_Si precipitates were broken into small particles and uniformly distributed in the matrix. This led to decreases in the interparticle distance and the average particle radius during the rolling process, and the ${∆\sigma }_{or}$ of the alloy increased after rolling process, further improving the strength of the LAS831 alloy.

(4) Load-transfer reinforcement

In duplex LAS831 alloys, the Mg_2_Si phase is stronger than the matrix phase. Figure 4e,f show that there is strong bonding in the interface between the strip-like Mg_2_Si and the matrix. Alloys containing banded reinforcing phases are similar to composite materials and, therefore, the influence of load transfer must be considered in the strength calculation. The contribution to the UTS of load-transfer reinforcement can be expressed as follows [38]:

$$∆\sigma_{lr}=\left(\sigma_{MS}-\sigma_{ML}\right)\times \lambda f_{MS}$$

where $\sigma_{MS}$ is the UTS of the Mg_2_Si phase, $\sigma_{ML}$ is the UTS of the matrix phase, *λ* is the correction coefficient, and $f_{MS}$ is the volume fraction of strip-like Mg_2_Si in the as-rolled LAS831 alloy. Because Mg_2_Si is much stronger than the matrix, a higher volume fraction of strip-like Mg_2_Si in the alloy results in a greater strengthening effect.

In addition to the above strengthening mechanism, the change in texture also affects the properties of Mg alloys [39,40,41,42]. Some research has shown that the texture affects the strength and plasticity of alloys, including duplex Mg–Li alloys [39,41,42], and the greater the texture strength, the more significant the improvement in alloy properties. The (0002) plane of the α-Mg phase produces a strong texture (maximum texture density of 10.3) in the duplex Mg–9Li alloy during plastic deformation processing, and the formation of strong (0002) basal texture of the α-Mg phase can greatly improve the strength and plasticity of the alloy [42]. Rolling deformation usually causes a change in grain orientation that produces texture, which increases with an increase in strain [43]. The deformation strain of LAS831 alloy increases with an increase in rolling passes, and the α-Mg phase texture strength of LAS831 alloy also increases. This is also one of the influencing factors that results in the strength of the LAS831 alloy significantly increasing, while the plasticity slightly decreases with the increase in rolling passes.

## 5. Conclusions

In this paper, the warm rolling process of LAS831 alloy with duplex-phase and the strengthen mechanism of the as-rolled LAS831 alloy were investigated. The microstructural evolution of the alloy was analyzed using optical microscopy (OM), transmission electron microscopy (TEM), and energy spectroscopy (EDS). Based on the experimental results, the following conclusions can be drawn:

(1) In the as-rolled LAS831 alloy, the highest ultimate tensile strength (323.66 ± 19.8 MPa) was obtained using a three-pass rolling process with a 30% thickness reduction per pass at 553 K. The strength of the alloy increases with an increasing number of rolling passes, but the elongation to failure decreases.
(2) The strength of the as-rolled LAS831 alloy is determined by a combination of second-phase strengthening, grain refinement strengthening, dislocation strengthening, and load-transfer reinforcement. Of these factors, dislocation strengthening caused by strain hardening of α-Mg phase can produce a good strengthening effect but also causes a decrease in plasticity.
(3) There is nanotransformation of the AlLi particles formed in the AlLi phase after three rolling passes; the fine Mg_2_Si and nanosized AlLi particles strengthen the second phase in forming a hard phase, and Mg_2_Si has greater second-phase strengthening ability than AlLi due to its high hardness and strong thermal stability.
(4) Because of its bcc structure, the β-Li phase has a high stacking fault energy, which is beneficial for dynamic recovery and dynamic recrystallization. The average size of the DRXed β-Li grains without dislocation tangles decreased with an increasing number of rolling passes, and the average size of recrystallized grains for three-pass-rolled LAS831 alloy became as low as 0.27 μm.
(5) The interface between the strip-like Mg_2_Si phase and the α-Mg phase is characterized by semicoherent bonding. This strong interface bonding can promote the easy transfer of tensile and shear forces applied on the matrix to the strip-like Mg_2_Si phase, thereby improving the matrix strength and strengthening the LAS831 alloy.

## Figures and Tables

**Table 1:** Actual chemical compositions of LAS831 alloys tested by ICP-OES.

Alloy	Elemental Composition (wt.%)			
	Li	Al	Si	Mg
LAS831	8.3	3.1	1.09	Bal

## Data Availability

The original contributions presented in the study are included in the article, further inquiries can be directed to the corresponding author.
